# Supplementary material for: Direct observation of translational activation by a ribonucleoprotein granule
Source: Nat Cell Biol. 2024 Jul 4;26(8):1322–35. doi: 10.1038/s41556-024-01452-5 (PMC11321996; doi:10.1038/s41556-024-01452-5)
Supplement: Supplementary file 1 — Supplementary Notes and Supplementary Fig. 1. [file 41556_2024_1452_MOESM1_ESM.pdf]

nature cell biology

Supplementary information https://doi.org/10.1038/s41556-024-01452-5

Direct observation of translational activation by a ribonucleoprotein granule

In the format provided by the authors and unedited

supplementary note

<u>Generation of CRISPR *suntag-nanos* line</u>

We made a substantial attempt to knock-in a SunTag array into the endogenous *nanos* locus using CRISPR-Cas9 genome editing. With the repair template and the two guide RNAs described in the method section, over 2000 embryos were injected by *BestGene*. Only one transformant was identified in the screen, which was used to establish the *suntag-nanos* line. In the repair template, the SunTag array was placed after the start codon of the Nanos open reading frame (ORF), and a DsRed marker cassette flanked by P-Bac transposon ends was put into the first intron of the *nanos* gene, which could be subsequently removed by P-Bac transposase. The sequencing of genomic DNA of the *suntag-nanos* line showed that the SunTag array was inserted into the correct position in the Nanos ORF. The DsRed marker cassette, however, was not in the first intron of *nanos*. Instead, the cassette, together with some flanking *nanos* sequences (parts of the left and right homology arms), was inserted upstream of *nanos* locus (about 300bp upstream of the transcription start site). This unexpected insertion may be the result of a rare splicing event of the repair template during homology-directed repair. In addition, we used different guide RNAs and/or different repair template designs (not presented in this paper), attempting to generate more transformant, but without success. As the SunTag was correctly inserted into the designed position in the *suntag-nanos* line, and our experiment validated that *suntag-nanos* mRNA showed a similar RNA localization and translation pattern as native *nanos* mRNA, we used this line throughout our study.

<u>Quantification of *suntag-nanos* mRNA translation</u>

The *suntag-nanos* line is homozygous-viable. Female flies homozygous for the *suntag-nanos* insertion lay a similar number of eggs as wildtype flies but the embryos did not hatch. The cuticles of embryos derived from homozygous females had either no or 1~3 abdominal segments (supplementary Table 4). This suggests that SunTag-Nanos can perform the function of Nanos in germline stem cells in the ovary but is unable to support posterior patterning in embryos. The insertion of the DsRed marker upstream of the *nanos* locus might disrupt an enhancer for *nanos* transcription because the mRNA abundance of the *suntag-nanos* RNA was significantly lower than that of the native *nanos* gene. The low expression, however, was advantageous for the quantification of the translating fraction. Under normal expression levels, *nanos* mRNAs form homotypic clusters, whereby each cluster contains multiple copies of *nanos* mRNA per germ granule. Thus, distinguishing between translating and non-translating mRNA using SunTag in a multi-copy mRNA cluster would have been technically challenging. The low expression of *suntag-nanos* reduces *nanos* mRNA levels to, on average, mostly one mRNA per granule (see the histogram below showing the quantification of RNA numbers in smFISH foci). This allowed us to quantify the translation of mostly single *suntag-nanos* mRNA per granule. Whether homotypic clustering of endogenous *nanos* mRNA in germ granules can affect the translation remains an open question and cannot be addressed with the lowly expressed *suntag-nanos* CRISPR line. A rigorous study on the effects of clustering requires a stronger expression

of *suntag-nanos*, which allows the analysis of the cluster with a higher amount of mRNA and a mathematical model to fit our quantification data.

Supplementary data

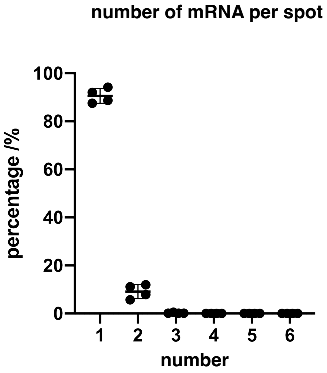

Supplementary figure 1. Distribution of the mRNA number per smFISH spot in germplasm. N=4 embryos.
