# Supplementary material for: Direct observation of translational activation by a ribonucleoprotein granule
Source: Nat Cell Biol. 2024 Jul 4;26(8):1322–35. doi: 10.1038/s41556-024-01452-5 (PMC11321996; doi:10.1038/s41556-024-01452-5)
Supplement: Supplementary file 2 — Reporting Summary [file 41556_2024_1452_MOESM2_ESM.pdf]

nature portfolio

Corresponding author(s): Ruth Lehmann

Last updated by author(s): May 20, 2024

Reporting Summary

Nature Portfolio wishes to improve the reproducibility of the work that we publish. This form provides structure for consistency and transparency in reporting. For further information on Nature Portfolio policies, see our Editorial Policies and the Editorial Policy Checklist.

Statistics

For all statistical analyses, confirm that the following items are present in the figure legend, table legend, main text, or Methods section.

n/a	Confirmed	
☐	☒	The exact sample size (*n*) for each experimental group/condition, given as a discrete number and unit of measurement
☐	☒	A statement on whether measurements were taken from distinct samples or whether the same sample was measured repeatedly
☐	☒	The statistical test(s) used AND whether they are one- or two-sided<br>*Only common tests should be described solely by name; describe more complex techniques in the Methods section.*
☒	☐	A description of all covariates tested
☐	☒	A description of any assumptions or corrections, such as tests of normality and adjustment for multiple comparisons
☐	☒	A full description of the statistical parameters including central tendency (e.g. means) or other basic estimates (e.g. regression coefficient) AND variation (e.g. standard deviation) or associated estimates of uncertainty (e.g. confidence intervals)
☐	☒	For null hypothesis testing, the test statistic (e.g. *F*, *t*, *r*) with confidence intervals, effect sizes, degrees of freedom and *P* value noted<br>*Give P values as exact values whenever suitable.*
☒	☐	For Bayesian analysis, information on the choice of priors and Markov chain Monte Carlo settings
☒	☐	For hierarchical and complex designs, identification of the appropriate level for tests and full reporting of outcomes
☐	☒	Estimates of effect sizes (e.g. Cohen's *d*, Pearson's *r*), indicating how they were calculated

Our web collection on statistics for biologists contains articles on many of the points above.

Software and code

Policy information about availability of computer code

Data collection	Zeiss ZEN 3.8
Data analysis	MatLab_R2021a, FIJI (ImageJ Version 2.14.0), ilastik (1.4.0b27), FISHQuant_v3, Colabfold (v1.5.2), IUPred2A, GraphPad Prism (8.4.3), Python3 and custom code run on python3 for image analysis. BioRender was used for creating figures. Custom Python scripts and ImageJ macro used to measure the distance between foci signals to granule borders have been uploaded to GitHub: https://github.com/wstainier/mRNA_distance_measurements.

For manuscripts utilizing custom algorithms or software that are central to the research but not yet described in published literature, software must be made available to editors and reviewers. We strongly encourage code deposition in a community repository (e.g. GitHub). See the Nature Portfolio guidelines for submitting code & software for further information.

Data

Policy information about availability of data

All manuscripts must include a data availability statement. This statement should provide the following information, where applicable:

- Accession codes, unique identifiers, or web links for publicly available datasets
- A description of any restrictions on data availability
- For clinical datasets or third party data, please ensure that the statement adheres to our policy

Previously published Oskar protein sequences from different Drosophila species that were re-analyzed here are available under accession codes NP_996186.1,

KAH8311831.1, XP_002053269.1, XP_023173869.2, XP_017140399.1, XP_001994345.1, XP_017968973.1, XP_001359508.2, XP_017856611.1, XP_002017385.1. Source data are provided within this paper. All other data supporting the findings of this study are available within the manuscript or can be obtained from the corresponding author on reasonable request.

Research involving human participants, their data, or biological material

Policy information about studies with human participants or human data. See also policy information about sex, gender (identity/presentation), and sexual orientation and race, ethnicity and racism.

Reporting on sex and gender	No human data in this study.
Reporting on race, ethnicity, or other socially relevant groupings	No human data in this study.
Population characteristics	No human data in this study.
Recruitment	No human data in this study.
Ethics oversight	No human data in this study.

Note that full information on the approval of the study protocol must also be provided in the manuscript.

Field-specific reporting

Please select the one below that is the best fit for your research. If you are not sure, read the appropriate sections before making your selection.

☒ Life sciences ☐ Behavioural & social sciences ☐ Ecological, evolutionary & environmental sciences

For a reference copy of the document with all sections, see nature.com/documents/nr-reporting-summary-flat.pdf

Life sciences study design

All studies must disclose on these points even when the disclosure is negative.

Sample size	No sample size calculation was performed. For the quantification of translation, 5-10 embryos were used for each genotype or condition; for the distance measurement, results from at least three embryos were combined; which we found sufficient to demonstrate the robustness and variability of the results based on preliminary experiments.
Data exclusions	No data has been systematically excluded.
Replication	All quantification experiments have been repeated at least once independently with similar results.
Randomization	Samples from wildtype (control) and mutant (experimental) fly strains were equally and randomly collected.
Blinding	Blinding was not done. Blinding was not possible when different groups can be easily identified during data collection (germplasm vs soma difference, wildtype vs mutant difference). Blinding was not necessay or practical during data analysis because all analysis was done using software automatically with the same setting.

Reporting for specific materials, systems and methods

We require information from authors about some types of materials, experimental systems and methods used in many studies. Here, indicate whether each material, system or method listed is relevant to your study. If you are not sure if a list item applies to your research, read the appropriate section before selecting a response.

Materials & experimental systems

n/a	Involved in the study	
☐	☒	Antibodies
☒	☐	Eukaryotic cell lines
☒	☐	Palaeontology and archaeology
☐	☒	Animals and other organisms
☒	☐	Clinical data
☒	☐	Dual use research of concern
☒	☐	Plants

Methods

n/a	Involved in the study	
☒	☐	ChIP-seq
☒	☐	Flow cytometry
☒	☐	MRI-based neuroimaging

Antibodies

Antibodies used	Commercial antibodies:rabbit anti-RPS6 (Cell Signaling #2217), rabbit anti-GCN4 (Novus Bio #C11L34 1:1000),ThermoFisher Scientific anti-rabbit AlexaFluor488 (A-11008), anti-rabbit AlexaFluor647 (A-31573) , anti-rat AlexaFluor555 (A-21434).<br>Lab-generated primary antibodies: rat anti-Oskar (gift from Paul Lasko) , rabbit anti-Nanos (Lehmann Lab), rabbit anti-CCR4 and anti-NOT3 (gift from Elmar Wahle)
Validation	Validations of commercial antibodies are described on manufacturers' websites:<br>rabbit anti-RPS6:https://www.cellsignal.com/products/primary-antibodies/s6-ribosomal-protein-5g10-rabbit-mab/2217?_requestid=3689689<br>rabbit anti-GCN4:https://www.novusbio.com/products/gcn4-antibody-c11l34_nbp2-81274<br>For anti-Oskar and anti-Nanos, we validated them by showing that the embryo staining results is consistent with the published localization of the proteins (Ephrussi and Lehmann, Nature. 1992 Jul 30;358(6385):387-92). Anti-CCR4 and anti-NOT3 have been validated by the published study: Temme et. al., RNA. 2010 Jul; 16(7): 1356–1370.

Animals and other research organisms

Policy information about studies involving animals; ARRIVE guidelines recommended for reporting animal research, and Sex and Gender in Research

Laboratory animals	Species: Drosophila melangaster; strains: w1118, yw, and derived lines. Age: 0-3h embryos.
Wild animals	The study did not involve wild animals.
Reporting on sex	The samples were eggs and ovaries collected from female flies. The sex of eggs were irrelevant and therefore not determined because processes we studied happen before the sex differentiation of the embryos.
Field-collected samples	The study did not invlove samples collected from the field.
Ethics oversight	No ethical oversight relevant.

Note that full information on the approval of the study protocol must also be provided in the manuscript.
